# Parathyroid Hormone 1 Receptor Signaling in Dental Mesenchymal Stem Cells: Basic and Clinical Implications

**DOI:** 10.3389/fcell.2021.654715

**Published:** 2021-10-25

**Authors:** Ping Lyu, Bo Li, Peiran Li, Ruiye Bi, Chen Cui, Zhihe Zhao, Xuedong Zhou, Yi Fan

**Affiliations:** ^1^State Key Laboratory of Oral Diseases, Department of Cariology and Endodontics, West China Hospital of Stomatology, National Clinical Research Center for Oral Diseases, Sichuan University, Chengdu, China; ^2^State Key Laboratory of Oral Diseases, Department of Orthodontics, National Clinical Research Center for Oral Diseases, West China Hospital of Stomatology, Sichuan University, Chengdu, China; ^3^State Key Laboratory of Oral Diseases, Department of Oral and Maxillofacial Surgery, National Clinical Research Center for Oral Diseases, West China Hospital of Stomatology, Sichuan University, Chengdu, China; ^4^Guangdong Province Key Laboratory of Stomatology, Guanghua School of Stomatology, Hospital of Stomatology, Sun Yat-sen University, Guangdong, China

**Keywords:** parathyroid hormone, parathyroid hormone-related peptide, regeneration, tooth development, periodontal ligament

## Abstract

Parathyroid hormone (PTH) and parathyroid hormone-related protein (PTHrP) are two peptides that regulate mineral ion homeostasis, skeletal development, and bone turnover by activating parathyroid hormone 1 receptor (PTH1R). PTH1R signaling is of profound clinical interest for its potential to stimulate bone formation and regeneration. Recent pre-clinical animal studies and clinical trials have investigated the effects of PTH and PTHrP analogs in the orofacial region. Dental mesenchymal stem cells (MSCs) are targets of PTH1R signaling and have long been known as major factors in tissue repair and regeneration. Previous studies have begun to reveal important roles for PTH1R signaling in modulating the proliferation and differentiation of MSCs in the orofacial region. A better understanding of the molecular networks and underlying mechanisms for modulating MSCs in dental diseases will pave the way for the therapeutic applications of PTH and PTHrP in the future. Here we review recent studies involving dental MSCs, focusing on relationships with PTH1R. We also summarize recent basic and clinical observations of PTH and PTHrP treatment to help understand their use in MSCs-based dental and bone regeneration.

## Brief Overview of Parathyroid Hormone 1 Receptor, Parathyroid Hormone, and Parathyroid Hormone-Related Protein

### Parathyroid Hormone 1 Receptor

PTH1R is one of the class B G-protein-coupled receptor (GPCR) family members with a seven-transmembrane structure (Sutkeviciute et al., 2019). PTH1R signaling plays a pivotal role in the regulation of multiple physiological functions including mineral ion homeostasis and skeletal development, as well as bone metabolism (Cheloha et al., 2015). It is widely expressed during early development but has its highest expression in bone and kidney (Urena et al., 1993). Upon binding with either of its two ligands, PTH and PTHrP, PTH1R is able to activate the Gα_*s*_/adenylyl cyclase/protein kinase A (PKA) pathway, Gα_*q*_/phospholipase C/protein kinase C (PKC) pathway, Gα_12/13_-phospholipase D/RhoA pathway as well as mitogen-activated protein kinase (MAPK) signaling cascade (Kondo et al., 2002; Singh et al., 2005; Syme et al., 2005; Gesty-Palmer et al., 2006; Sneddon and Friedman, 2007).

### Parathyroid Hormone

PTH is an 84 amino-acid (AA) endocrine hormone secreted by the parathyroid glands and serves as the mediator of extracellular calcium and phosphate levels and skeletal homeostasis (Wein and Kronenberg, 2018).

#### Parathyroid Hormone 1 Receptor Signaling in Mineral Ion Metabolism

The metabolism of calcium and inorganic phosphate is tightly controlled by the orchestration of several key organs: parathyroid gland, intestine, kidney, and bone. PTH is secreted from parathyroid glands and plays a critical role in controlling hormonal and cellular responses that regulate mineral ion homeostasis. PTH restores serum calcium levels by three distinct actions. In the kidneys, PTH stimulates the expression of 25-hydroxyvitamin D-1α-hydroxylase, which leads to increased 1,25(OH)_2_D synthesis (Bergwitz and Jüppner, 2010). It also promotes Ca^2+^ reabsorption in the distal tubules in the kidney (Boros et al., 2009). In bone, PTH has been identified as a crucial mediator of bone-formation and bone-resorption to release calcium and phosphate from the matrix into the bloodstream (Jilka, 2007; Yang et al., 2007). In the intestine, PTH-induced 1,25(OH)2D increases dietary absorption of calcium. Regarding phosphate maintenance, PTH functions to inhibit Napi2a and Napi2c expressions in the luminal brush border membrane of proximal tubules cells in the kidney, thereby inhibiting phosphate reabsorption (Picard et al., 2010). Moreover, it induces fibroblast growth factor 23 (FGF23) production in osteoblasts and osteocytes (Rhee et al., 2011b; Meir et al., 2014; Fan et al., 2015). FGF23 functions to suppress phosphate reabsorption and reduce calcitriol synthesis (Shimada et al., 2004; Ohnishi et al., 2009). Therefore, systemic mineral ion balance largely depends on PTH, 1,25(OH)_2_D, and FGF23 acting in cooperation.

#### The Role of Parathyroid Hormone in Bone Remodeling

PTH plays a key role in bone remodeling by targeting skeletal stem cells, bone marrow stromal cells, osteoprogenitors, osteoclasts, bone lining cells, osteocytes, osteoclast, and T lymphocytes/macrophages (Rhee et al., 2011a; Wein and Kronenberg, 2018). The anabolic effect of PTH on the skeleton is well-established in osteoporosis (Neer et al., 2001; Black et al., 2008). Intermittent PTH (iPTH) administration stimulates new bone formation by directing mesenchymal stem cell fate and activating bone lining cells, as well as promoting the activity and differentiation of osteoblasts (Kim et al., 2012; Fan et al., 2017). It can also suppress the apoptosis of mature osteoblasts and osteocytes as well as decrease sclerostin expression (Jilka et al., 1999; Keller and Kneissel, 2005; Jilka, 2007; Delgado-Calle et al., 2017). On the other hand, sustained elevations of PTH levels by hyperparathyroidism or continuous PTH administration results in loss of bone mass due to excessive bone resorption through the production of receptor activator for nuclear factor-κB ligand (Rankl) in PTH-targeted cells (Bilezikian et al., 2018; Delgado-Calle et al., 2018).

### Parathyroid Hormone-Related Protein

PTHrP can exert its physiological functions by acting as a paracrine, autocrine, or intracrine mediator in multiple target tissues. It has a similar N-terminal amino acid sequence to PTH (1–34) and binds the same receptor, PTH1R (Sutkeviciute et al., 2019). The first 13 residues of PTHrP show the highest degree of primary sequence homology with PTH, 8 of which are identical (Suva et al., 1987). This region is important for almost all the agonist effects of PTHrP and PTH. The PTHrP 14–36 region has little or no sequence homology with PTH but is needed for binding to PTH1R and activating subsequent signaling cascades (Juppner et al., 1991). Additionally, the 36–139 AA region of PTHrP contains unique functional domains. For instance, PTHrP residues 35–84 are responsible for placental calcium transport (Abbas et al., 1989), while the 107–139 region can promote osteoblast proliferation and function while inhibiting osteoclast activity (Fenton et al., 1993; Cornish et al., 1997, 1999; Alonso et al., 2008). PTHrP has context-dependent effects in multiple tissues, including the growth plate, bone, placenta, blood vessel, skin, and tooth (Hirai et al., 2015; Martin, 2016). It is primarily involved in embryonic skeleton development and postnatal bone formation, as well as in placental calcium mobilization during gestation and lactation (Neville et al., 2002; Kronenberg, 2006; McCauley and Martin, 2012). In the growth plate, PTHrP is secreted locally by chondrocytes, and has a pivotal function in endochondral bone formation during development (Lanske et al., 1996; Vortkamp et al., 1996). A population of skeletal stem cell is identified in PTHrP-positive chondrocytes within the resting zone of a growth plate (Mizuhashi et al., 2018). PTHrP interacts with Indian hedgehog (Ihh) secreted from the hypertrophic zone and delays the differentiation toward hypertrophic chondrocytes (Vortkamp et al., 1996). Deletion of the gene encoding PTHrP (*Pthlh*) causes chondrocyte hypertrophy on the anterior rib cartilage, thus leading to early lethality in mutants due to respiratory failure (Karaplis et al., 1994). Overexpression of human PTHrP protein in collagen type II-expressing cells rescued the chondrocyte hypertrophy phenotype, suggesting that PTHrP synthesized by and secreted from chondrocytes functions to inhibit chondrocyte hypertrophy in the growth plate (Weir et al., 1996). Furthermore, PTHrP-haplo-insufficient mice display low bone mass owing to decreased recruitment of precursor cells concomitant with accelerated osteoblast apoptosis (Amizuka et al., 1996). In osteoblasts, specific *Pthlh* deletion leads to osteoporosis and impaired bone formation (Miao et al., 2005). In osteocytes, PTHrP acts in a paracrine/autocrine manner to induce bone formation and to modulate adult cortical bone strength (Ansari et al., 2018). These data indicate that PTHrP may duplicate the anabolic effects of iPTH in osteoblastic cells.

In addition to the many past studies that focus on the role of PTH1R signaling in mineral ion metabolism and skeletal homeostasis (Fan et al., 2015; Carrillo-López et al., 2019; Fu et al., 2020; Martin et al., 2021), interest has recently turned to its function in craniofacial development and remodeling. The major elements of the oral cavity, including teeth, periodontal tissues, and the jaw bones (maxilla and mandible), are intimately connected. Teeth develop in jaw bones and are closely connected to alveolar bone through periodontal tissues. Thus, researchers have embarked on efforts to understand the regulatory network of signaling pathways in teeth and adjacent tissue. As a result, PTH1R signaling has been found to exert profound influence in the orofacial region (Frazier-Bowers et al., 2014). PTH1R signaling is involved in tooth eruption, tooth root formation, alveolar bone regeneration, and periodontium repair (Aggarwal and Zavras, 2012; Ono et al., 2016; Nagata et al., 2020; Zhang et al., 2020). Dental mesenchymal stem cells (MSCs) are key targets for PTH1R signaling in these physiological activities. This emphasizes the need to understand both MSCs and the underlying mechanisms that regulate them. Here, we review the recent literature for regulatory networks involved with PTH1R signaling in mediating MSCs with a particular focus on orofacial MSCs. Furthermore, there is growing knowledge of the therapeutic application of PTH and PTHrP analogs in dental implant surgery, orthodontics, periodontitis, and orofacial regeneration medicine. This review discusses these advances and provides a summary of the key functions of PTH1R signaling on MSCs in dental tissue homeostasis and repair.

## Parathyroid Hormone 1 Receptor Signaling and Bone Marrow-Derived Mesenchymal Stem Cells

MSCs can be isolated from many tissues, including bone marrow, circulating blood, placenta, cord blood, synovial tissue, pancreas, skeletal muscle, adipose tissue, and oral and maxillofacial tissue (Vaananen, 2005). The self-renewal capacity and potential plasticity of MSCs make them indispensable for organ development and tissue repair (Bianco et al., 2013). Beginning with Friedenstein’s elegant experiments, much work has been focused on bone marrow mesenchymal stem cells (BMMSCs) (Friedenstein et al., 1974a,b). PTH1R signaling is an important regulator of the ontogeny of bone marrow, its stroma and the BMMSCs residing in it (Kuznetsov et al., 2004). For instance, PTH acts directly on cultured bone marrow-derived stromal cells to increase their proliferation and differentiation (Nishida et al., 1994). *In vivo* lineage tracing experiments showed that intermittent administration of PTH (1–34) upregulated Nestin-positive BMMSCs (Mendez-Ferrer et al., 2010). Similarly, the number of cells expressing Sox9 significantly increased upon PTH (1–34) treatment. Moreover, these cells differentiated into osteoblasts more quickly than those in vehicle-treated mice (Balani et al., 2017). Also, PTH has been shown to target a subset of leptin receptor (LepR) expressing cells to increase the expression of runt-related transcription factor 2 (Runx2), a transcription factor that is required for osteogenesis, and promotes the differentiation of these stem cells (Ducy et al., 1997; Yang et al., 2017). More recently, we and others have described the function of PTH1R signaling on mesenchymal cell fate decision. PTHrP-haplo-insufficient mice have low bone mass and increased marrow adiposity (Amizuka et al., 1996). Ablation of PTH1R in Prx1-positive MSCs results in decreased bone formation, increased bone marrow adipose tissue, and accelerated bone resorption (Fan et al., 2017). This is likely due to the downstream cascade of PTH1R signaling through Gα_*s*_ since loss of Gα_*s*_ in osteoprogenitors results in a similar phenotype (Sinha et al., 2014). Additionally, iPTH treatment shifted the differentiation of LepR-positive progenitor cells from an adipo-lineage toward an osteo-lineage, accompanied by higher expression of osteogenic markers and reduced adipocyte markers (Yang M. et al., 2019). These data emphasize the central role of PTH1R signaling in guiding BMMSCs toward an osteoblast lineage and away from adipogenesis.

PTH1R, in conjunction with different downstream pathways, acts in BMMSCs to drive various biological activities. The mechanisms and the therapeutic potential of this activity are of great interest. Studies suggest that PTH (1–34) enhances the migration and adhesion of BMMSCs through Rictor/mTORC2 signaling *in vitro* (Lv et al., 2020). Additionally, PTH has been shown to influence and expand the bone marrow stem cell niche (Huber et al., 2014). iPTH treatment was found to enhance stem cell homing via an SDF-1α/CXCR4 axis (Huber et al., 2014). Further study focusing on the therapeutic application of PTH showed that iPTH improved i.v. MSC therapy to promote bone loss healing by inducing the migration of MSCs to defective sites (Sheyn et al., 2016). Moreover, PTH can be combined with a scaffold to act as a biomaterial to induce bone regeneration by enhancing osteogenesis of BMMSCs via Notch signaling (Zou et al., 2021).

## Parathyroid Hormone 1 Receptor Signaling Regulates Dental Mesenchymal Stem Cells

**FIGURE 1:**
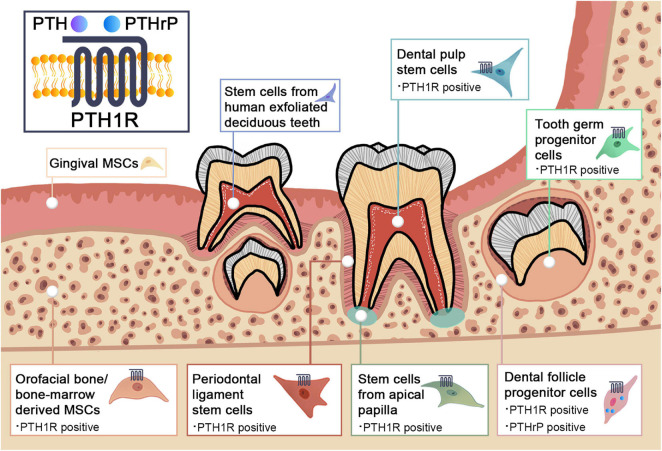
Schematic graph illustrating the PTH1R signaling in dental mesenchymal stem cell populations.

There is accumulating evidence for a link between PTH1R signaling and MSCs. In addition to bone marrow, orofacial tissues are one of the major sources of MSCs. There are a variety of stem cell populations that can be identified in teeth and their supporting structures, including dental pulp stem cells (DPSCs), stem cells from human exfoliated deciduous teeth (SHEDs), periodontal ligament stem cells (PDLSCs), dental follicle progenitor cells (DFPCs), stem cells from apical papilla (SCAPs), orofacial bone/bone-marrow-derived MSCs (OMSCs), tooth germ progenitor cells (TGPCs), and gingival MSCs (GMSCs) (Zheng et al., 2019). These stem cells express distinct surface markers and have multi-lineage differentiation capacities with region-specific characteristics. Figure 1 and Table 1 illustrate the regulatory function of PTH1R signaling in dental MSCs.

**TABLE 1:** Features of dental tissue derived MSCs and the regulation role of PTH1R signaling.

	Origins	Surface markers		Lineage tracing animal models	Differentiation capabilities		Effect of PTH1R signaling
					
		+	−		*In vitro*	*In vivo*	
Dental pulp stem cells (DPSCs)	Dental pulp	CD13, CD29, CD44, CD73, CD90, CD105, CD106, CD146, CD166, CD271, STRO-1, STRO-3	CD3, CD8, CD14, CD15, CD19, CD33, CD34, CD45, CD71, CD117, CD133	Wnt1, Gli1, α-SMA, Prx1	Osteogenic, chondrogenic, adipogenic, chondrogenic, neurogenic, hepatogenic, cardiogenic, endothelial cells, pancreatic cells	Form dentin-pulp like complex Regenerate pulp Repair peripheral nerve injury	Enhanced osteo/odontogenic differentiation Stimulated phosphorylation of PKB/AKT, ERK, JNK
Dental follicle progenitor cells (DFPCs)	Dental follicle	CD29, CD44, CD73, CD90, CD105, CD146, Notch1, STRO-1, Nestin	CD14, CD31, CD34, CD45, CD117	Osx, PTHrP, Gli1, Prx1	Osteogenic, adipogenic, chondrogenic, cementogenic, neurogenic	Regenerate periodontal tissue Root regeneration	Regulated the differentiation of Osx^+^ and PTHrP^+^ progenitors during tooth root formation PTHrP in epithelium stimulated DFPCs-induced osteoclast formation
Orofacial bone/bone-marrow-derived MSCs (OMSCs)	Orofacial bone/bone-marrow	CD29, CD44, CD73, CD90, CD105, CD106, SSEA-4, Oct-4	CD14, CD19, CD31, CD34, CD45	Wnt1, LepR, Prx1	Osteogenic, adipogenic, chondrogenic	Form bony tissue	Increased osteogenesis of Prx1^+^ progenitors during eruption Enhanced proliferation and osteogenesis of LepR^+^ progenitors Downregulated p16ink4a and specific senescent-associated secretory phenotype in aged OMSCs
Periodontal ligament stem cells (PDLSCs)	Periodontal ligament	CD29, CD44, CD73, CD90, CD105, CD146, CD166, STRO-1, STRO-4	CD14, CD31, CD34, CD40, CD45, CD54, CD79a, CD80, CD86	Lrig1, Wnt1, Axin2, Gli1	Adipogenic, chondrogenic, osteogenic, neurogenic, cementogenic, cardiogenic, pancreatic cells, ectoderm lineage cells	Form cementum-PDL like structure Regenerate periodontal tissue	Regulated PDL formation, construction of collagen fibers, and Periostin expression Induced osteogenesis of STRO1^+^ PDLSCs Assisted SDF-1α in recruiting PDLSCs to periodontal defects
Stem cells from apical papilla (SCAPs)	Apical papilla	CD24, CD29, CD73, CD90, CD105, CD106, CD146, CD166, STRO-1	CD14, CD18, CD34, CD45, CD150	Wnt1, Gli1	Odontogenic, osteogenic, adipogenic, chondrogenic, neurogenic	Form root-periodontal like complex	Enhanced osteo/odontogenic differentiation Stimulated phosphorylation of ERK, JNK
Tooth germ progenitor cells (TGPCs)	Third molar tooth germ	CD29, CD44, CD73, CD90, CD105, CD166	CD14, CD34, CD45, CD133, CD117	N/A	Odontogenic, osteogenic, adipogenic, neurogenic, endothelial cells, epithelial cells	Contribute to neuro protection	PTH (1–34) enhanced osteogenic ability at early developmental stage PTH (53–84) stimulate osteogenic ability at late stage
Stem cells from human exfoliated deciduous teeth (SHEDs)	Human exfoliated deciduous teeth	CD13, CD29, CD44, CD90, CD105, CD146, STRO-1	CD3, CD14, CD19, CD34, CD106, HLA-DR	N/A	Osteogenic, odontogenic, adipogenic, neurogenic, endothelial-genic	Form bony tissue Regenerate pulp Regenerate periodontal tissue Promote facial nerve repair	N/A
Gingival mesenchymal stem cells (GMSCs)	Gingiva	STRO-1, CD29, CD44, CD73, CD90, CD105, CD146	CD11b, CD34, CD45, HLA-DR	N/A	Osteogenic, chondrogenic, adipogenic, keratogenic	Form bony tissue	N/A

### Dental Pulp Stem Cells

Dental pulp stem cells (DPSCs) are the first dental MSCs to be identified. They are highly proliferative with multilineage differentiation potential including osteogenic and chondrogenic capability (Gronthos et al., 2000). DPSCs, derived from dental pulp tissues by enzymatic digestion, are able to form a dentin-pulp-like complex when transplanted into immunocompromised mice (Gronthos et al., 2002). They participate in dental repair processes and play an important role in stem cell-based therapy for endodontic regeneration (Shi et al., 2020). Several studies have revealed the applicability of DPSCs in regeneration of the dentin-pulp complex and the repair of peripheral nerve injury (Lambrichts et al., 2017).

Recent studies have implicated PTH1R signaling in mediating DPSCs function. However, PTH treatment did not alter the proliferation rate of DPSCs. Rather, the expression levels of osteo/odontogenic related genes were significantly upregulated along with an increase in the formation of mineralized nodules and calcium content. This suggests that PTH has a positive effect on DPSCs differentiation (Ge et al., 2020). MAPKs are involved in DPSCs proliferation and differentiation, so researchers have explored the roles of extracellular signal-regulated kinase (ERK) and P38 MAPK pathways in PTH-treated DPSCs (Wang B.L. et al., 2019; Yu et al., 2019; Ge et al., 2020). PTHrP also enhances odontogenic differentiation of dental pulp cells by stimulating phosphorylation levels of protein kinase B (PKB/AKT), ERK, and c-Jun N-terminal kinase (JNK), all essential molecules of the ERK and P38 MAPK pathways (Kim et al., 2020). Immunohistochemistry analysis revealed that PTHrP is only present in a few fibroblasts and cells of the odontoblastic zone in normal pulps, whereas the distribution of PTHrP markedly expands in inflamed pulps (Marigo et al., 2010). PTHrP-positive cells were found in the vascular zone, pulp stroma, and the odontoblastic and sub-odontoblastic zones. This suggests that PTHrP may contribute to angiogenesis during inflammation (Marigo et al., 2010). However, further studies are needed to elucidate the underlying mechanisms of PTH1R signaling in regulating DPSCs.

### Dental Follicle Progenitor Cells

These MSCs are found in the dental follicle (DF), a mesenchymal condensation structure surrounding the developing tooth germ prior to eruption. DFPCs are progenitor cells of periodontal tissues with the ability to form PDL, cementum, and alveolar bone during tooth development (Morsczeck et al., 2005). Like DPSCs, DFPCs are multipotent cells with the capability to differentiate into osteoblasts, chondrocytes, cementoblasts, and adipocytes, as well as neuronal cells (Yao et al., 2008; Zhou et al., 2019). DFPCs are responsible for the development of cementum, periodontal ligament, and alveolar bone during tooth eruption and tooth root morphogenesis (Zhou et al., 2019). Strong evidence indicates that PTH1R signaling plays a crucial role during tooth eruption (Izumida et al., 2020). Genetic studies have revealed that familial primary failure of tooth eruption (PFE) is a PTH1R-associated hereditary disease (Frazier-Bowers et al., 2014). It is causally linked to heterozygous mutations of PTH1R (Frazier-Bowers et al., 2014). More studies are focusing on characterization of the biological role of PTH1R in the dental follicle during tooth eruption. These studies suggest that mesenchymal progenitor cell populations reside in the DF, including cells expressing Osterix (Osx), PTHrP, and Gli1 (Liu et al., 2015; Ono et al., 2016; Takahashi et al., 2019). This information was used to generate conditional knockout of PTH1R based on these markers in order to explore its mechanism in DF progenitors. Ablation of PTH1R in Osx^+^ progenitors resulted in failed tooth eruption with significantly truncated roots that lack periodontal ligaments and ankylosis of the dental root (Ono et al., 2016). PTH1R-deficient progenitors also showed impaired proliferation ability and accelerated differentiation into cementoblasts. This was accompanied by upregulation of the bone/cementum matrix protein osteopontin (Opn) and nuclear factor I/C (Nfic), leading to an unusual formation of cellular cementum on the root surface (Ono et al., 2016). The ability of Nfic to enhance proliferation and differentiation of odontoblasts was verified *in vitro*. Also, Nfic deficiency led to truncation of molar roots (Steele-Perkins et al., 2003; Lee et al., 2009). The detailed mechanism of how PTH1R signaling regulates Nfic requires further exploration. Interestingly, knocking out histone deacetylace-4 (HDAC4) partially recapitulated the phenotypes in *OsxCre;PTH1R^*fl/fl*^* mice such as short root and thicker cementum, indicating that HDAC4 might be a key mediator downstream of PTH1R signaling in DFPCs (Ono et al., 2016).

PTHrP, one of the ligands of PTH1R, is a required paracrine/autocrine cytokine in regulating DFPCs (Nagata et al., 2020). Earlier research identified PTHrP as a secretory factor in epithelial components, functioning as a paracrine molecule mediating epithelial-mesenchymal interactions during development (Kitahara et al., 2002). PTHrP is present in the enamel organ (Suda et al., 2003) and is responsible for the formation of the eruption pathway (Philbrick et al., 1998). This observation has inspired various studies that focused on the role of PTHrP in mediating osteoclastogenesis (Wise et al., 2000). An experiment was conducted in which PTHrP activity was inhibited with antiserum and conditional media in PTHrP-treated dental follicle cells to induce bone resorption in fetal-rat long bone. This work by Nakchbandi et al. (2000) showed that PTHrP was required for osteoclast formation and differentiation. Coculture experiments with DFPCs and epithelial stellate reticulum cells indicated that PTHrP secreted by epithelial cells could stimulate DFPCs-induced osteoclast formation, contributing to bone resorption on the coronal aspect of an erupting tooth (Nakchbandi et al., 2000). In addition, PTHrP has effects beyond its paracrine functions. A recent study using lineage tracing experiments discovered the presence of PTHrP in a group of DFPCs where it acted as an essential autocrine ligand modulating the physiological cell fates of DFPCs through PTH1R (Takahashi et al., 2019). PTH1R deficiency in PTHrP^+^ DFPCs leads to failure of tooth eruption accompanied by loss of periodontal attachment and abnormal cellular cementum formation. This is mainly due to a shifting of the cell fate to non-physiological cementoblast-like cells in association with higher expression of bone/cementum matrix protein and a crucial transcription factor Mef2c (Takahashi et al., 2019). Additionally, PTHrP simulated osteogenesis of human DFPCs cells independently of Ihh in an autocrine manner (Klingelhoffer et al., 2016). Furthermore, PTHrP has been observed to have intracrine actions as well (Fiaschi-Taesch and Stewart, 2003). It is reported that a dental follicle progenitor cell line with higher endogenous expression of PTHrP had increased alkaline phosphatase (ALP) activity and the ability to induce mineralization by activating the bone morphogenetic protein (BMP) signaling pathway (Pieles et al., 2020). The intracrine mode of PTHrP was discovered with the observation that intranuclear endogenously expressed PTHrP and its function are not affected by external PTHrP or PTH1R inhibitors (Pieles et al., 2020).

In addition to PTH and PTHrP, other molecules can mediate DFPCs through PTH1R signaling. Recent work indicates that PTH1R expression correlated with that of chromodomain helicase DNA-binding protein 7 (CHD7), a chromatin remodeling enzyme. Upregulation of CHD7 significantly accelerated osteogenic differentiation of human dental follicle cells while knockdown of CHD7 gave the reverse result. The latter was partially rescued by overexpression of PTH1R (Liu C. et al., 2020). Others have noted that PTH1R signaling potentially interacts with several key signaling pathways such as Wnt/β-catenin, Hedgehog (Hh), and TGF-β/BMP in DFPCs during tooth eruption (Nagata et al., 2020), yet the underlying mechanisms remain to be determined.

### Orofacial Bone/Bone Marrow-Derived Mesenchymal Stem Cells

The common sources of BMMSCs are the femur and tibia, but MSCs have also been isolated from alveolar bone marrow (Matsubara et al., 2005; Miura et al., 2006). These are termed orofacial bone/bone marrow-derived MSCs (OMSCs) (Yamaza et al., 2011). They share some features with long bone or iliac bone-derived BMMSCs. However, OMSCs exhibit distinct characteristics in terms of higher proliferation, higher expression of ALP, and more calcium accumulation *ex vivo* (Akintoye et al., 2006). OMSCs have shown promising potential for bone regeneration, especially in the orofacial region owing to the proximate embryonic origin and microenvironment (Lee et al., 2019).

Zhou et al. (2014) recently discovered that a small proportion of bone marrow cells express LepR, a marker highly enriched in BMMSCs. These cells play a major role in bone formation, bone repair upon injury, and adipo-differentiation in adult bone marrow (Zhou et al., 2014). Research with LepR^+^ MSCs in the alveolar region verified that LepR labels a population of OMSCs which are quiescent physiologically under normal conditions but contribute to intramembranous bone formation when activated by external stimulation such as tooth extraction. Moreover, intermittent PTH (1–34) treatment increased the number of LepR^+^ cells in the alveolar ridge and promoted osteogenic differentiation of LepR^+^ cells. In contrast, mice that lack PTH1R in LepR^+^ cells showed impaired socket repair after tooth extraction, highlighting the crucial role of PTH1R signaling in LepR^+^ OMSCs during regeneration of jawbone defects (Zhang et al., 2020).

Prx1 is another marker for craniofacial mesenchyme and Prx1-positive progenitors are located at the base of molars and alveolar bone marrow surrounding incisors (Logan et al., 2002; Cui et al., 2020a). Mice with conditional knockout of PTH1R in Prx1^+^ cells had arrested tooth eruption, decreased alveolar bone formation and downregulated osteogenesis-related genes. Consistent with *in vivo* analyses, PTH1R-deficient OMSCs showed decreased ALP and alizarin red (ARS) staining concomitant with reduced expression of osteogenic related markers Osx, Runx2, Osteocalcin (Ocn), and Dentin matrix protein 1 (Dmp1). There was also reduced expression of downstream factors of the BMP/TGF-β pathway, including Tgfbr1, Tgfb1, and Bmp1. These data suggest that PTH1R signaling mediates tooth eruption by regulating osteogenic differentiation of OMSCs in alveolar bone, which contributes to the motive force during eruption (Cui et al., 2020a).

PTH1R signaling also plays a crucial role in aging and senility related changes and diseases (Cui et al., 2020b). Intermittent PTH (1–34) treatment may function to lower the number of membrane TGF-β receptors that then suppress the expression of p-Smad3 (Crane and Cao, 2014), a molecule can accelerate secretion of senescence biomarker p16ink4a. The suppression of p-Smad3 leads to downregulation of p16ink4a and alleviation of the specific senescent-associated secretory phenotype (SASP) in OMSCs. This creates a microenvironment with decreased senescent cell burden. These factors provide a favorable treatment path for aging/senility related diseases (Cui et al., 2020b).

### Periodontal Ligament Stem Cells

PDLSCs were identified in the periodontal ligament (PDL), the soft tissue that links cementum of roots to the alveolar bone (Fleischmannova et al., 2010). PDLSCs contribute to periodontal tissue formation *in vivo* and generate a cementum/PDL-like structure *in vitro* (Seo et al., 2004). PDLSCs exhibit multi-lineage differentiation potential. They can give rise to adipocytes, chondrocytes, osteoblasts, and cementoblast-like cells under certain stimuli (Seo et al., 2004). Research showed that PDLSCs, together with scaffolds, promote PDL, cementum, and alveolar bone formation at periodontal injury sites with bone defects (Iwasaki et al., 2019). The evidence indicates that PDLSCs contribute to periodontal tissue repair and support the great potential of PDLSCs for promoting tissue regeneration and reconstructing the connection between the alveolar socket and tooth root in periodontitis (Hernández-Monjaraz et al., 2018).

A variety of studies using animal models have demonstrated the importance of PTH1R signaling in regulating stem cells that reside in the PDL. Cui et al. (2020a) recently revealed that PDL cells from a mouse incisor originated from Prx1^+^ progenitors. Lack of PTH1R expression resulted in a narrowed PDL with irregularly aligned collagen fibers, downregulated Periostin expression, and aberrant formation of bone-like tissue in PDL (Cui et al., 2020a). In the same vein, conditional ablation of PTH1R in Osx-lineage cells resulted in a thinner, less organized PDL in *OsxCre;PTH1R^fl/+^* mice and complete loss of the PDL with ankylosed root in homozygous knockout mice (Ono et al., 2016). These studies provide a deeper insight into PTH1R signaling in PDL development during tooth root formation and the eruption process.

### Stem Cells From Apical Papilla

The apical papilla is a transient zone located at the apices of immature permanent teeth and is related to root formation (Gan et al., 2020). SCAPs have higher proliferative ability than DPSCs and can give rise to odontoblastic/osteoblastic cells, adipocytes, and neural progenitor-like cells under certain stimulation (Sonoyama et al., 2008; Songsaad et al., 2020). Several discoveries have shed light on the function of SCAPs in root formation and tooth eruption. SCAPs have the ability to induce continued root maturation and have been used to treat interrupted root formation caused by periradicular periodontitis or abscess (Huang et al., 2008). Moreover, a recent study suggests the promising application of SCAPs in pulp regeneration and bioengineered tooth root (bio-root) engineering (Gan et al., 2020).

Pang et al. (2020) administered intermittent PTH (1–34) treatment to SCAPs and noted an increase in ALP activity and expression of odonto/osteogenic markers, including Ocn, Opn, Osx, Runx2, Col1αI, and dentin sialophosphoprotein (DSPP). Yet the proliferation of SCAPs was unchanged upon PTH administration. It was shown that iPTH administration upregulated p-JNK in SCAPs during the first 15 min, and induced p-P38 in 30 min while downregulating p-ERK. This is consistent with another finding that dephosphorylating ERK induced osteogenesis differentiation and bone formation (Huang et al., 2007). It is important to note that p-ERK was increased in PTH-treated DPSCs in conjunction with osteo-differentiation enhancement, suggesting that the nature of the crosstalk between PTH1R signaling and JNK, P38 MAPK pathways could be different in specific stem cell lineages (Ge et al., 2020).

### Tooth Germ Progenitor Cells

Tooth germ is comprised of enamel organ, dental papilla, and dental follicle. Through the sequential and mutual epithelial-mesenchymal interactions, tooth germ gradually developed into enamel, dentin, pulp, and surrounding supportive tissues. A novel population of MSCs referred to as TGPCs was discovered in the late bell stage tooth germ of the third molar. Since tooth germ is at an early developmental stage with both mesenchymal and epithelial original tissues, TGPCs were able to differentiate into odontogenic, osteogenic, adipogenic, and neurogenic cells, as well as endothelial-, epithelial-like cells (Yalvaç et al., 2010; Taşlı et al., 2013, 2014; Doğan et al., 2015). Moreover, they have a neural protection function by increasing antioxidant enzymes and reducing neuronal death or apoptosis (Yalvaç et al., 2013). TGPCs have stable stem cell properties and present more immature features compared with other dental MSCs. Resulted from the wide range of indications for third molar extractions, TGPCs are easily accessed with minimal invasiveness, implying promising therapeutic applications of TGPCs in tooth regeneration in the future (Yalvaç et al., 2010).

Previous research reported the effects of distinct fragments of PTH on tooth germ development at different stages. Administration of PTH (1–34) to tooth germ from mouse embryos resulted in upregulated ALP activity at an early developmental stage while downregulated ALP occurred at a late stage. In contrast, the action of another PTH fragment, PTH (53–84) exerted opposite effects (Tsuboi and Togari, 1998). Further investigation is required to understand the temporospatial effect of distinct PTH fragments in mediating tooth germ development.

### Stem Cells From Human Exfoliated Deciduous Teeth

SHEDs were first isolated from the pulp tissue of the crowns of exfoliated deciduous teeth (Miura et al., 2003). SHEDs are characterized as immature DPSCs expressing embryonic stem cell markers. They show multidirectional differentiation potential and higher proliferation capacity when compared with adult MSCs such as DPSCs and PDLSCs (Miura et al., 2003). *In vivo* transplantation of SHEDs demonstrated that they could induce bone formation and promote facial nerve regeneration (Pereira et al., 2019). Implanted SHEDs could survive in mouse brain and express neural markers (Miura et al., 2003). They are also promising candidates for dental pulp tissue engineering (Rosa et al., 2016). The capacity of odontogenic differentiation makes them an alternative source for stem cell-mediated bio-root regeneration (Yang X. et al., 2019). Combined with treated dentin matrix, SHEDs can successfully regenerate periodontal tissues, including PDL, blood vessels, and alveolar bone (Yang X. et al., 2019). Obtained from primary teeth, SHEDs are easily accessed by a minimally invasive procedure. Thus, SHEDs have drawn great and long-lasting attention since they were first discovered (Miura et al., 2003). Recent research reported that FGF2 enhanced the angiogenesis, osteogenesis, and proliferation of SHEDs (Novais et al., 2019). FGF signaling is involved in directing osteo/odontogenic differentiation of SHEDs by regulating phosphate/pyrophosphate regulatory genes (Nowwarote et al., 2018). Another highlight of SHEDs is their participation in physiological root resorption during the eruption process of permanent teeth. SHEDs can promote osteoclastogenesis, which is driven by tumor necrosis factor-α (TNF-α) through NF-κB signaling (Wang C. et al., 2019). It remains uncertain whether PTH1R signaling has a function in regulating SHEDs. Considering the resemblance and tight connections between SHEDs and DPSCs, it is worth exploring the underlying regulation mechanisms related to PTH1R signaling in various biological properties and tissue engineering applications of SHEDs.

### Gingival Mesenchymal Stem Cells

Gingiva, a crucial component of tooth adjacent soft tissue, is another source of dental MSCs. GMSCs exhibit self-renewal and multilineage differentiation capacity (Tang et al., 2011). GMSCs lack tumorigenicity and can give rise to osteoblasts, chondroblasts, adipocytes, and keratinocytes (Santamaría et al., 2017; Murugan Girija et al., 2018). Several *in vivo* studies confirmed the ability of GMSCs in bone defects regeneration (Al-Qadhi et al., 2021). The osteogenic capability of GMSCs can be stimulated by proinflammatory cytokines and stress response proteins during inflammation (Tomasello et al., 2017). It is reported that GMSCs could suppress osteoclastogenesis and ultimately bone resorption via the CD39-adenosine pathway, suggesting a therapeutic application of GMSCs for rheumatoid arthritis and other related diseases (Luo et al., 2019). Thus, GMSCs can be a promising candidate for the repair and regeneration of inflammation- related bone loss, such as periodontitis and periapical infectious diseases. The advantage of GMSCs in treating inflammatory diseases can be attributed to their ability in regulating immune responses. It has been reported that exosomes from TNF-α treated GMSCs induced M2 macrophage polarization, therefore suppressing inflammation and periodontal bone loss (Nakao et al., 2021). Another study demonstrated the function of GMSCs in treating graft-vs.-host disease (GVHD) through mediating the conversion of Tregs to Th1 and/or Th17-like cells (Ni et al., 2019). Moreover, evidence shows that GMSCs display a more stable morphology and retain MSC features at higher passages compared to BMMSCs (Tomar et al., 2010). STRO-1 may be a useful marker to evaluate the stem cell properties of GMSCs since its expression will gradually decrease as the passage number increases. There is a paucity of data relative to PTH1R signaling in mediating GMSCs. Further study will help to understand the mechanisms for modulating these MSCs and widen the potential use of these MSCs in therapeutic applications.

### Limitations and Future Directions

A combination of studies has revealed the involvement of PTH1R signaling in regulating various types of dental MSCs. Progress has been made in understanding the downstream molecules and pathway networks, but there are several aspects that have not been fully elucidated. First, there are overlapping regulatory mechanisms active in different dental MSCs. PTH1R signaling promotes osteo/odontogenic differentiation of both DPSCs and SCAPs through MAPK pathways. Increased Runx2, Osx, and Ocn expression were found in both OMSCs and SCAPs upon PTH1R activation. Whether there are stem cell-specific targeting factors or pathways remains to be determined. Second, administrating PTH or PTHrP to dental MSCs may interact with multiple signaling pathways, including Wnt, Hh, and TGF-β. For instance, it has been reported that PTH stimulates bone formation in osteoblasts and osteocytes, partially through canonical Wnt signaling (Tobimatsu et al., 2006; Suzuki et al., 2008; Robling et al., 2011; Bellido et al., 2013). Details of the crosstalk among these signaling cascades in dental MSCs are of interest and need to be analyzed in future studies. Third, current studies mainly focus on PTH1R signaling in mediating the properties of dental MSCs in normal physiological states. Whether there are diverse regulatory mechanisms involved under pathological conditions remains a subject of further research.

Furthermore, *Cre* transgenic mouse models revealed that diverse lineages of dental MSCs orchestrate and then contribute to craniofacial development under regulation by PTH1R signaling. However, there are still several limitations regarding the utilization of conditional knock-out mouse models. First, there is lack of specific *Cre* mouse models targeting MSCs residing in dental tissues. Many of the studies involving the orofacial region were inspired by research focusing on long bone, including the choice of transgenic *Cre* animal models. For instance, conditional knockout of PTH1R is not restricted to craniofacial area using *Osx-Cre*, *PTHrP-Cre*, or *Prx1-Cre*. Whole skeleton *Cre* recombinase knockout may affect mineral ion homeostasis or whole-body metabolism, which subsequently influences craniofacial development. Therefore, specific mouse models targeting dental MSCs are required for further investigation. Second, some *Cre* mouse models induce defects in the craniofacial region during the embryonic stage. For example, *Osx-Cre* mice display slight growth delay and intramembranous bone hypomineralization (Wang et al., 2015a) and this may cause overlapping phenotypes and interference in the craniofacial region. Therefore, it is critical to select Cre-positive littermates as controls when analyzing the effect of targeted deletion of a floxed gene. Third, mouse models that depend on Cre recombinase result in embryonic deletion of target genes. All of the daughter cells inherit the same inactivated gene, which may lead to an overestimation in the function of gene or cell population. Complete loss of the proximal mandibular arch was observed in *Nestin-Cre*;*Fgf8^*fl/fl*^* mice (Trumpp et al., 1999). Although this phenotype proved the significance of Fgf8 in the development of the mandible, it held back further investigations on the tissue specific function of Fgf8 (Trumpp et al., 1999). Moreover, some embryonic knockouts may raise the mortality in mutant mice (Lei et al., 2016; Ono et al., 2016). Therefore, Cre^ER^ system is a more desirable strategy since it enables the choice of the timepoint for triggering the knockout. To date, many of the PTH1R conditional ablation models focused on the craniofacial developmental stage. Inducible Cre mouse models will enable an understanding of the function of PTH1R in adulthood. For example, although the function of PTH1R in DFPCs has been comprehensively characterized during root development, the postnatal role of PTH1R of PDLSCs in PDL homeostasis and injury repair remains to be determined. When applying a Cre^ER^ strategy, it is important to ascertain the heterogenicity of different subsets in specific start points and dosage of the drug delivery when inducing the Cre recombinase. The development of single-cell technology may favor dissection of the subsets and their lineage allocation (Nagata et al., 2020). It may also lead to discovery of new marker genes specific to dental MSCs, which provides clues for generating novel inducible transgenic lines to directly target specific cell types.

## Therapeutic Application of Parathyroid Hormone/Parathyroid Hormone-Related Protein in Orofacial Region

Multiple stem cell populations reside in teeth and their supporting tissues. Many that are regulated by PTH1R signaling have been identified, indicating the potential to use PTH/PTHrP to facilitate stem-cell-based oral tissue engineering and regeneration. The positive effects of PTH and PTHrP on vertebral and appendicular bone mineral density have been well-characterized. Teriparatide is a recombinant form of human PTH (1–34) and was the first anabolic drug approved by the U.S. Food and Drug Administration (FDA) to treat osteoporosis (Us Food and Drug Administration, 2002). A second anabolic agent, abaloparatide (ABL), is the 1–34 analog of PTHrP and was approved in the U.S. and Canada (Health Canada, 2017; Us Food and Drug Administration, 2017). Teriparatide and ABL both act through PTH1R on bone cells to stimulate intracellular cAMP and subsequent gene expression that favors bone formation (Yang et al., 2007). Both can increase bone mineral density while reducing the probability of fracture (Rosen, 2004; Miller et al., 2016; Ramchand and Seeman, 2020). Recent studies of ABL suggested that it has greater potential to widen the bone anabolic window and alleviate side effects compared to teriparatide (Makino et al., 2021). ABL contains modifications in AA residue insertions to maximize its anabolic capacity (Tella et al., 2017). It can be further differentiated from teriparatide based on its affinity and selectivity for PTH1R R^*G*^ (Dean et al., 2008). This property allows more transient cAMP signaling to increase osteogenesis, thus exerting more prominent anabolic potential (Hattersley et al., 2016). Moreover, endogenous PTH has a catabolic function in bone, while the incidence of hypercalcemia and increased 1,25(OH)_2_D_3_ is lower in treatment with ABL when compared to teriparatide (Miller et al., 2016). Low resorptive actions makes ABL a new generation of anabolic drugs with a promising future (Ardura et al., 2019).

Several studies have subsequently investigated the effects of PTH and PTHrP on oral and maxillofacial bone regeneration (Rowshan et al., 2010; Valderrama et al., 2010). Indeed, alveolar bone is the major target of iPTH treatment in periodontitis, osteonecrosis of the jaw, and jawbone defects (Bashutski et al., 2010; Kuroshima et al., 2013; Sim et al., 2020). In addition, iPTH has been used to enhance bone-implant osseointegration and bone remodeling in orthodontic treatment (Nimeri et al., 2013; Jiang et al., 2018). The function of PTHrP has also been explored in dental implant treatment. In this section, we will briefly summarize the recent applications of PTH and PTHrP in the orofacial region.

### Lesion of Periodontal Tissue

A variety of studies using both animal models and clinical trials have suggested a promising outcome for PTH treatment in periodontitis. PTH-treated periodontitis rodents had reduced alveolar bone resorption and milder infiltration of inflammatory cells at the marginal gingiva (Barros et al., 2003; Chen et al., 2017). PTH applied to rats with partially removed PDL and cementum attenuated the extension of the bone defect, increased total callus bone, and accelerated the formation of cementum-like tissue at the healing site (Vasconcelos et al., 2014). Moreover, local injection of PTH, or PTH in combination with neutral self-assembling peptide hydrogel, could improve clinical outcomes of chronic periodontitis (Tokunaga et al., 2011; Yoshida et al., 2019). A pre-clinical study conducted on type 1 diabetic rats with periodontitis demonstrated that iPTH treatment downregulates sclerostin, the Wnt signaling inhibitor, and stimulates osteoid formation and mineralization (Kim et al., 2018). Importantly, the most notable clinical example is a double-blind, placebo controlled, randomized trial involving the application of PTH to 40 patients undergoing periodontal surgery. In this study, patients receiving teriparatide showed higher gain in bone height, improved periodontal attachment, and better outcome of a periodontal probing examination (Bashutski et al., 2010).

The therapeutic potential of PTH (1–34) for periodontitis may depend on PDLSCs. Evidence shows that PTH (1–34) treatment, together with osteogenic induction, conducted on STRO-1^+^ human PDLSCs led to increased expression of Runx2 and Osx, along with upregulated mineralization ability. It is worth noting that PTH1R expression increased in hPDLSCs upon PTH treatment, implying that a positive feedback loop was established (Wang X. et al., 2016). Stromal cell-derived factor-1α (SDF-1α) is a key factor in stem cell recruitment and homing in many diseased organs requiring regeneration (Ceradini et al., 2004). Yet its therapeutic potential is limited by CD26/dipeptidyl peptidase-IV (DPP-IV) which leads to N-terminal cleavage at the position-2 proline of SDF-1α (Christopherson et al., 2004). PTH is a DPP-IV inhibitor and therefore can assist SDF-1α in recruiting PDLSCs to the injured site (Huber et al., 2014). Furthermore, since SDF-1α and PTH can both promote proliferation and osteogenic differentiation of PDLSCs, PTH/SDF-1α co-therapy becomes a promising strategy for periodontitis (Du et al., 2016). An *in vivo* study confirmed that PTH/SDF-1α co-therapy could induce chemotaxis of CD90^+^CD34^–^ stromal cells and stimulate their migration toward periodontal defects, concomitant with increased expression of Runx2, ALP, and Col1αI in the newly formed bone area. The result is accelerated bone regeneration and better organization of the periodontal ligament interface (Wang F. et al., 2016).

### Bisphosphonate-Related Osteonecrosis of the Jaws

Bisphosphonates (BPs), the first line drugs used to treat osteoporosis, act by suppressing osteoclasts to achieve anti-resorptive activity (Black and Rosen, 2016). Bisphosphonate-related osteonecrosis of the jaws (BRONJ), a rare but severe complication, is characterized by non-healing jaw defects in patients who have undergone BP treatment. The etiology remains unclear, but one hypothesis is that BPs inhibit bone turnover in skeletal homeostasis and healing processes (Grey, 2010; Rollason et al., 2016). Considering the anabolic and catabolic effects of PTH, off-label use of teriparatide has been applied to BRONJ treatment with promising outcomes in several clinical trials (Cheung and Seeman, 2010; Yoshiga et al., 2013; Kim et al., 2014; Jung et al., 2017; Sim et al., 2020). It is reported that teriparatide administration was associated with a higher resolution rate of necrosis lesions, reduced bone defects, and improved bone healing (Kim et al., 2014; Sim et al., 2020). Most trials conducted daily PTH injection. Whether weekly administration will have satisfactory therapeutic effects is unclear and remains under further investigation (Yoshiga et al., 2013; Ohbayashi et al., 2020). An animal model showed that in addition to BRONJ treatment, PTH also has potential to prevent the development of jaw necrotic lesions by maintaining osteocyte survival (Kuroshima et al., 2014a). The administration of teriparatide led to a higher ratio of RANKL-positive osteocytes (Liu J. et al., 2020). Another study that applied different doses of teriparatide suggested that its therapeutic effect may not be dose dependent (Yu and Su, 2020). Further investigation of the dosage, frequency, duration of treatment, and drug combination plan for PTH treatment in BRONJ is warranted.

### Dental Implant

iPTH treatment is critical for osteogenesis at the peri-implant area and for osseointegration at the external and internal surfaces of implants in aged rats and in osteoporosis animal models induced by ovariectomy, glucocorticoid, or low protein diet (Dayer et al., 2010; Almagro et al., 2013; Oki et al., 2017; Park et al., 2017; Jiang et al., 2018). It is worth noting that treating jaw osteoporosis with PTH (1–34) before implant surgery restored bone quality, bone volume, and bone turnover, leading to favorable implant stability (Gomes-Ferreira et al., 2020). Furthermore, PTH (1–34) can be used to reverse the deleterious effects of cigarette smoke such as poor bone healing and low bone mass in the bone-implant interface (Lima et al., 2013). In cases where BRONJ was induced by dental implants, PTH also enhanced peri-implant bone formation (Park et al., 2020). However, a few studies suggest that iPTH does not improve healing of the augmented maxillary sinus in osteoporosis rabbit models or osteointegration in diabetic rats (Rybaczek et al., 2015; Dam et al., 2020). Local delivery systems have been implemented by binding PTH to polyethylene glycol (PEG)-based hydrogel or by directly depositing PTHrP on implants using a layer-by-layer (LBL) electro assembly technique. The therapeutic efficacy of both systems is promising (Valderrama et al., 2010; Tang et al., 2020). In addition, a pre-clinical trial that combined PTH with other factors such as bisphosphonates, menaquinone-4 (vitamin K2; MK), or simvastatin showed a cumulative advantage (Li et al., 2013, 2018; Tao et al., 2016). Moreover, there is evidence that withdrawal of PTH after the course of treatment reversed the effects and bone mineral density decreased rapidly. However, anti-resorptive therapy such as bisphosphonates applied after iPTH could restore the implant anchorage and maintain the curative effect (Hu et al., 2019). Mechanical loading is another factor that contributes to peri-implant bone mass, but whether the combination of loading and PTH has an additive effect requires further investigation (Fahlgren et al., 2013; Grosso et al., 2015; Shibamoto et al., 2018). In a clinical trial, Kuchler et al. (2011) conducted an open-label, placebo-controlled, randomized trial of 24 patients with edentulous mandibles to investigate the therapeutic effects of teriparatide administration on dental implant osseointegration. The results revealed that 20 μg of teriparatide administered once-per-day for 28 days increased bone to implant contact as well as bone formation in the periosteal, cortical, and medullary compartment (Kuchler et al., 2011).

### Extraction Socket Healing

Osteoporosis delayed extraction socket healing in a pre-clinical study (Liu et al., 2019). iPTH therapy through subcutaneous or intra-oral injection were both effective in promoting healing in the tooth extraction socket by increasing new bone formation and connective tissue maturation (Kuroshima et al., 2013). In teriparatide treated osteoporosis rats, the extraction socket exhibited increased expression levels of osteogenic markers including Wnt, Alp, and Ocn and reduced osteoclast numbers (de Oliveira et al., 2019). In comparison with bisphosphonates, PTH has a better therapeutic effect in both hard and soft tissue regeneration at the extraction site by enhancing osteogenesis and collagen deposition while suppressing inflammation (Kuroshima et al., 2014b). However, PTH did not have a significant effect on extraction wound healing in hyperglycemic rats (Xu et al., 2020). Another study also demonstrated that PTH did not benefit the healing process of the bone-implant interface in diabetic rats (Rybaczek et al., 2015). However, PTH treatment had a positive effect on periodontitis in a diabetic rodent model (Kim et al., 2018). These conflicting results indicate that whether diabetes exerts an influence on the effect of PTH requires further investigation.

### Autograft/Allograft Bone Integration

The success of autograft or allograft treatment for massive craniofacial bone defects is limited by rapid resorption, poor integration, and scar formation (Burchardt, 1983; Cohn Yakubovich et al., 2017a). PTH can augment new bone formation, as well as reduce bone resorption and fibrotic tissue formation in grafts harvested from calvaria or iliac (dos Santos et al., 2016; Zandi et al., 2019), improving reconstruction of bone defects with autografts. Allografts face the challenge of integrating with the host bone. However, PTH is reported to stimulate osteoprogenitor differentiation and enhance bone formation and bone mineralization in calvaria or mandible defects (Sheyn et al., 2013; Pelled et al., 2020). It promotes neovascularization in the graft surroundings (Cohn Yakubovich et al., 2017a), resulting in less fibrosis and scar tissue formation as well as superior allograft integration.

### Radiation-Induced Bone Injury

Radiotherapy for tumors in the orofacial area may cause radiation-induced bone injury such as decreased bone density and strength, or osteoradionecrosis in the jawbone. The main reasons for these issues are radiation-induced cell apoptosis and impaired regeneration ability (Smith et al., 2017). iPTH administration has been shown to rescue radiation-induced osteoblast apoptosis by enhancing DNA repair through the Wnt signaling pathway (Chandra et al., 2015). Moreover, PTH functions to maintain osteocyte count and enhance bone regeneration, thus alleviating radiation-induced bone loss (Chandra et al., 2014; Kang et al., 2017). The bone union quality was improved with increased trabecular number, thickness, and connectivity upon PTH treatment, indicating that PTH can rescue irradiated microstructural deterioration (Chandra et al., 2013; Gallagher et al., 2013).

### Orthodontic Treatment

Studies in rodents have suggested a function for PTH in accelerating orthodontic tooth movement (Nimeri et al., 2013). Research shows that iPTH accelerated tooth movement in osteoporosis rats and rabbits receiving mandibular ramus osteotomy (Salazar et al., 2011; Li et al., 2019). In terms of retention, iPTH could promote periodontium regeneration to reduce the relapsing distance, therefore maintaining the stability of orthodontic treatment (Li et al., 2020). Its ability to enhance periodontal tissue repair following orthodontic movement may be associated with suppression of high mobility group box protein 1 (HMGB1) (Wolf et al., 2013). In addition, PTH mediated cementum formation possible by regulating Dmp1 expression via the cAMP/PKA pathway in cementoblasts, offering a promising method to prevent root resorption during orthodontic treatment (Wang et al., 2015b; Li et al., 2021).

### Temporomandibular Joint Related Diseases

There has been progress made in exploring the effect of iPTH administration in the temporomandibular joint (TMJ). iPTH increases cell proliferation and differentiation in mandibular condylar cartilage (MCC), increasing the length of the condyle head (Mh et al., 2017). However, PTH could also exert negative effects on MCC by inducing early mineralization, leading to cartilage degeneration, and condyle surface irregularities (Dutra et al., 2017). We have recently generated an age-related TMJ osteoarthritis (TMJ OA) mouse model and injected them with PTH (1–34) for 4 weeks. The results suggest that iPTH ameliorated the degenerative changes in TMJ condyles and increased subchondral bone turnover by accelerating the differentiation of stem cells residing in the subchondral bone (Cui et al., 2020b). A recent study using a canine total meniscal meniscectomy model suggested PTH (1–34) with a BMMSCs-loaded scaffold stimulated meniscus regeneration and alleviated cartilage damage. This implies that PTH could promote the regenerative and chondroprotective function of a BMMSCs scaffold (Zhao et al., 2020). These data yield insight into the efficacy of combining PTH and stem cells in TMJ tissue engineering in the future.

### Fracture Healing and Distraction Osteogenesis

PTH (1–34) augments MSCs injection to treat fractures by enhancing MSCs homing and differentiation (Cohn Yakubovich et al., 2017b). It also enhances angiogenesis in bone callus (Jiang et al., 2019). Conversely, lack of endogenous PTH led to reduced vessel formation and downregulation of the PKA/pAKT/HIF1α/VEGF pathway during fracture healing (Ding et al., 2018). Distraction osteogenesis is a surgical technique for lengthening the bone, but rapid distraction may lead to poor bone healing (Ye et al., 2017). PTH functions to promote new bone formation and improve bone microarchitecture in the distracted callus (Ye et al., 2017). PTH also has positive effects in distraction of an irradiated mandible by reversing poor vascularity induced by radiation (Kang et al., 2013).

### Clinical Concerned Problems and Future Directions

Large numbers of pre-clinical animal studies and clinical trials have confirmed the therapeutic effect of PTH in the orofacial region. However, there are some limitations associated with PTH administration. Evidence from a single pre-clinical study using a rat model suggested long-term use and overdoses of teriparatide increased the risk of osteosarcoma (Vahle et al., 2002). The duration of teriparatide treatment is limited to 2 years to avoid this possibility (Hodsman et al., 2005). The contraindications for PTH injection include patients sensitive to osteosarcoma, patients with Paget’s disease, pre-existing hypercalcemia, and history of other skeletal disorders (Hodsman et al., 2005). Moreover, drug combination therapy and sequential therapy need further exploration since the therapeutic effect of PTH decreases rapidly after withdrawal (Black et al., 2005). It is reported that anti-RANKL antibodies and bisphosphonates may be therapeutic options for the discontinuation of iPTH treatment (Omiya et al., 2018; Hu et al., 2019).

PTH administration methods consist of systemic injection, local injection, dissolving in an injectable gel to form a local slow-release system and others (Soma et al., 2000). Several new methods have been developed to provide more convenient and effective ways to apply PTH or PTHrP as well as to reduce side effects. Among the various delivery methods proposed are an implantable and biodegradable pulsatile device (Dang et al., 2017), bio-membrane fabrication (Yin et al., 2020), and a nanoemulsion system (Altaani et al., 2020). Most of the current research has not progressed beyond the pre-clinical phase, thus detailed guidance for dosage, frequency, duration, and clinical indication of PTH or PTHrP administration in orofacial region is still required. So far, ABL-associated pre-clinical research and clinical trials targeting the orofacial region are limited. Furthermore, the role of other engineered amino-terminally modified variants of PTH, such as long-acting PTH (Shimizu et al., 2016) has not been analyzed in orofacial tissue. Further investigation is required to completely explore the theoretical basis of PTH/PTHrP analogs and its application in orofacial related diseases.

## Conclusion

A large and diverse number of studies have shed light on the role of PTH1R signaling in dental MSCs, emphasizing the important regulatory role of PTH1R signaling during development and pointing out the therapeutic potential of PTH and PTHrP analogs for dental tissue regeneration. The stem cell properties of a diverse family of dental MSCs have been well characterized in the past two decades. However, the underlying mechanisms for PTH1R signaling to mediate stem cell behavior are still uncertain. Many of the regulatory mechanisms have been demonstrated by *in vitro* experiments that suggest PTH1R signaling promotes proliferation and differentiation of multiple dental progenitors. The recent genetic tools of lineage tracing and conditional knock-out mouse models have helped to identify the specific population of MSCs regulated by PTH1R. Remarkably, pre-clinical studies and clinical trials have already revealed promising outcomes when administrating PTH or PTHrP in various dental-related diseases, such as periodontitis, osteonecrosis of the jaw, and jawbone defects. It is conceivable that activating PTH1R signaling has an effect on tissue resident MSCs during the process of repair. Yet a better understanding is necessary for a holistic view of the *in vivo* regulatory mechanisms of PTH1R in specialized cell types. In the development of stem-cell-based regeneration medicine, therapy based on PTH1R signaling plus dental MSCs holds great promise.

## Author Contributions

PiL, BL, PeL, RB, CC, and YF collected the literature and drafted the manuscript. ZZ, XZ, and YF supervised the procedures and approved the manuscript. All authors gave their final approval and agreed to be accountable for all aspects of the work.

## Conflict of Interest

The authors declare that the research was conducted in the absence of any commercial or financial relationships that could be construed as a potential conflict of interest.

## Publisher’s Note

All claims expressed in this article are solely those of the authors and do not necessarily represent those of their affiliated organizations, or those of the publisher, the editors and the reviewers. Any product that may be evaluated in this article, or claim that may be made by its manufacturer, is not guaranteed or endorsed by the publisher.
